# Temporal ChIP-on-Chip of RNA-Polymerase-II to detect novel gene activation events during photoreceptor maturation

**Published:** 2010-02-17

**Authors:** Padmaja Tummala, Raghuveer S. Mali, Eduardo Guzman, Xiao Zhang, Kenneth P. Mitton

**Affiliations:** Eye Research Institute, Oakland University, Rochester, MI

## Abstract

**Purpose:**

During retinal development, post-mitotic neural progenitor cells must activate thousands of genes to complete synaptogenesis and terminal maturation. While many of these genes are known, others remain beyond the sensitivity of expression microarray analysis. Some of these elusive gene activation events can be detected by mapping changes in RNA polymerase-II (Pol-II) association around transcription start sites.

**Methods:**

High-resolution (35 bp) chromatin immunoprecipitation (ChIP)-on-chip was used to map changes in Pol-II binding surrounding 26,000 gene transcription start sites during photoreceptor maturation of the mouse neural retina, comparing postnatal age 25 (P25) to P2. Coverage was 10–12 kb per transcription start site, including 2.5 kb downstream. Pol-II-active regions were mapped to the mouse genomic DNA sequence by using computational methods (Tiling Analysis Software-TAS program), and the ratio of maximum Pol-II binding (P25/P2) was calculated for each gene. A validation set of 36 genes (3%), representing a full range of Pol-II signal ratios (P25/P2), were examined with quantitative ChIP assays for transcriptionally active Pol-II. Gene expression assays were also performed for 19 genes of the validation set, again on independent samples. FLT-3 Interacting Zinc-finger-1 (FIZ1), a zinc-finger protein that associates with active promoter complexes of photoreceptor-specific genes, provided an additional ChIP marker to highlight genes activated in the mature neural retina. To demonstrate the use of ChIP-on-chip predictions to find novel gene activation events, four additional genes were selected for quantitative PCR analysis (qRT–PCR analysis); these four genes have human homologs located in unidentified retinal disease regions: Solute carrier family 25 member 33 *(Slc25a33),* Lysophosphatidylcholine acyltransferase 1 *(Lpcat1),* Coiled-coil domain-containing 126 *(Ccdc126),* and ADP-ribosylation factor-like 4D *(Arl4d).*

**Results:**

ChIP-on-chip Pol-II peak signal ratios >1.8 predicted increased amounts of transcribing Pol-II and increased expression with an estimated 97% accuracy, based on analysis of the validation gene set. Using this threshold ratio, 1,101 genes were predicted to experience increased binding of Pol-II in their promoter regions during terminal maturation of the neural retina. Over 800 of these gene activations were additional to those previously reported by microarray analysis. *Slc25a33*, *Lpcat1*, *Ccdc126*, and *Arl4d* increased expression significantly (p<0.001) during photoreceptor maturation. Expression of all four genes was diminished in adult retinas lacking rod photoreceptors (*Rd1 mice*) compared to normal retinas (90% loss for *Ccdc126* and *Arl4d*). For rhodopsin (*Rho*), a marker of photoreceptor maturation, two regions of maximum Pol-II signal corresponded to the upstream rhodopsin enhancer region and the rhodopsin proximal promoter region.

**Conclusions:**

High-resolution maps of Pol-II binding around transcription start sites were generated for the postnatal mouse retina; which can predict activation increases for a specific gene of interest. Novel gene activation predictions are enriched for biologic functions relevant to vision, neural function, and chromatin regulation. Use of the data set to detect novel activation increases was demonstrated by expression analysis for several genes that have human homologs located within unidentified retinal disease regions: *Slc25a33*, *Lpcat1*, *Ccdc126*, and *Arl4d.* Analysis of photoreceptor-deficient retinas indicated that all four genes are expressed in photoreceptors. Genome-wide maps of Pol-II binding were developed for visual access in the University of California, Santa Cruz (UCSC) Genome Browser and its eye-centric version EyeBrowse (National Eye Institute-NEI). Single promoter resolution of Pol-II distribution patterns suggest the *Rho* enhancer region and the *Rho* proximal promoter region become closely associated with the activated gene’s promoter complex.

## Introduction

Cell-specific gene expression is required for the specialized functions of an adult tissue. Depending on the tissue of interest, we may know about hundreds of the relevant genes and even several key transcription factors that regulate their expression. Unfortunately, our knowledge is not complete regarding the number of genes that must become active or their identities. Chromatin immunoprecipitation (ChIP)-on-chip analysis of RNA polymerase-II (Pol-II) binding in gene promoters has revealed additional and novel gene activation changes in cultured cells, including embryonic stem cells and cancer cells [1]. We decided to adapt this analysis to the neural retina, with the goal of finding additional genes that are activated during terminal maturation of photoreceptors in the mouse.

The mammalian neural retina is an established model to study both the differentiation and maturation of neurons [2]. Six major neuronal cell types and one glial cell type are generated from multipotent progenitors during development. For this study, we focused on a window of mammalian retinal development, which occurs between postnatal day 2 and 25 (P2 and P25) in the mouse. This phase starts from a point when the lineage of most cells is already decided but many genes expressed in the adult retina are not yet active, including those required for phototransduction, synaptogenesis, and neuron function. Several well studied photoreceptor-specific genes, such as rhodopsin (*Rho*), are activated during this final phase of development and also provide excellent internal markers for a temporal ChIP-on-chip study.

Regulation of gene expression certainly involves transcriptional initiation when DNA-binding transcription factors recruit chromatin-regulating (epigenetic) proteins and the general transcriptional machinery. Gene expression may also be regulated during transcript elongation or degradation [3-5]. Guenther et al. used Pol-II ChIP-on-chip of embryonic stem cells to show that Pol-II is detectable downstream of promoters that produce detectable transcripts [6]; they also reported many quiescent gene promoters that were preloaded with inactive Pol-II. We have previously demonstrated, by quantitative ChIP, that active Pol-II is present in the *Rho* gene’s transcriptional region only once the gene is active in the maturing neural retina [7,8]. We do not know if preloading of quiescent gene promoters with Pol-II occurs before their activation in the adult retina. Pol-II ChIP-on-chip mapping around transcription start sites would also shed light on this question.

We mapped Pol-II binding in mouse gene promoter regions, comparing P2 and P25 neural retina, using ChIP-on-chip with a high-resolution (35 bp) mouse promoter tiling array. Active regions of Pol-II binding were correlated to specific genes, and the Pol-II peak signal ratio (P25/P2) was used to predict activation increases during terminal maturation. Prediction accuracy was established by additional analyses of 36 genes, including quantitative ChIP (qPCR) analysis for transcribing Pol-II downstream of the transcription start site (TSS) and gene-expression assays (mRNA). In addition to Pol-II, we mapped FLT-3 Interacting Zinc-finger (FIZ1) recruitment to gene promoter complexes. While FIZ1 expression is ubiquitous in adult mammals, FIZ1 protein content increases tenfold in postnatal mouse retina [8]. FIZ1 is a zinc-finger protein that is recruited to several promoter complexes of photoreceptor genes, which are expressed during terminal maturation of the neural retina [7,8]. We used this basic property of FIZ1 as an additional marker for photoreceptor genes that are particularly activated during this final portion of development.

Pol-II ChIP-on-chip predicted that 863 genes would experience the largest proportion of their activation after the P2 developmental time point. These genes represent key biologic processes, such as gene regulation, signal transduction, chromatin organization, epigenetic regulation, visual transduction, neural development, ion transport, and synaptic transmission. Many known retinal disease genes involved in photoreceptor function and the regulation of photoreceptor genes were included. Of these genes 28% also contained significant FIZ1 active regions. Hundreds of the activated genes detected by ChIP-on-chip were unique determinations when compared to two previous expression-array studies. Many of the genes have human homologs located in mapped regions of unidentified retinal diseases.

In addition to providing a global analysis for gene activation, the high-resolution Pol-II ChIP-on-chip data revealed interactions of regulatory regions within the *Rho* promoter. While ChIP-on-chip analysis is most often used on cell cultures, our results demonstrate that this method can be applied to a complex neural tissue. Pol-II maps were also adapted for graphical exploration and sharing using the University of California, Santa Cruz (UCSC) Genome Browser and the eye-centric genome browser at National Eye Institute/National Institutes of Health (NEI/NIH; EyeBrowse).

## Methods

### Animal care and use

All animal care and tissue collections performed in this study were performed with the approval of Oakland University’s Animal Care and Use Committee (Rochester, MI), and conformed to the USA Department of Agriculture standards and to the Association for Research in Vision and Ophthalmology statement for the use of animals in ophthalmic and vision research. C57BL/6 and *Rd1* mice were obtained from Charles River Laboratories (Wilmington, MA) and housed at Oakland University in a facility approved by the Association for Assessment and Accreditation of Laboratory Animal Care International.

### Tissue isolation and chromatin cross-linking

C57BL/6 mice were the source for normal neural retina at P2 and P25 and were processed as previously described for ChIP PCR [7]. Older mice (P25, P33) were sacrificed using a CO_2_ chamber, and P2 mice by cervical dislocation. Retinas were dissected from eyes in PBS (137 mM NaCl, 97 mM Na_2_HPO_4_, 25 mM NaH_2_PO_4_,  26 mM KCl, Fisher Scientific, Pittsburg, PA), drained of excess PBS, and fast frozen on dry ice. For both P2 and P25 ages, the retinas were dissected working under a stereo dissection microscope to ensure tissues were free of optic nerve, retinal pigment epithelium, and the ciliary body. Care was also taken to remove any remnants of the tunica vasculosa lentis from P2 neural retinas. To obtain sufficient chromatin for ChIP-on-chip, 46 retinas were pooled for P2 and 60 retinas were pooled for P25. More tissue was required with P25 retinas, which yield less chromatin than P2 retinas. Frozen neural retina tissue was kept at −80 °C until protein/chromatin cross-linking with freshly prepared formaldehyde (1% w/v, Sigma Chemicals, St. Louis, MO) for 15 min at room temperature, with agitation. Cross-linking was stopped by addition of 0.125 M glycine (final concentration), and tissue was disrupted with a Dounce homogenizer. Lysates were then sonicated and the DNA sheared to an average length of 300–500 bp. Genomic DNA (input) was prepared by treating aliquots of chromatin with RNase, proteinase K, and heat (65 °C) to reverse cross-linking, followed by phenol and chloroform extractions and ethanol precipitation. Pellets were resuspended in 10 mM Tris, 1 mM EDTA, and the resulting DNA was quantified on a Nanodrop spectrophotometer (Thermo Fisher Scientific, Chicago, IL). Results were used to calculate the total chromatin yield.

### Chromatin Immunoprecipitation

For ChIP, an aliquot of chromatin (20 µg) was precleared with protein-A agarose beads (Invitrogen, Carlsbad, CA). Pol-II binding was detected using an antibody qualified for ChIP (sc-9001, Santa Cruz Biotechnology, Santa Cruz, CA). FIZ1 was detected using a polyclonal antibody specific for the FIZ1 protein, anti-GST-bFIZ1 (Proteintech Group Inc., Chicago, IL), which was previously qualified for quantitative ChIP analysis [7]. After incubation at 4 °C overnight, protein-A beads were used to isolate the immune complexes. Complexes were washed, eluted from the beads with sodium dodecyl sulfate (SDS) wash buffer, and subjected to RNase and proteinase-K treatment. Cross-links were reversed by incubation overnight at 65 °C, and ChIP DNA was purified by phenol-chloroform extraction and ethanol precipitation. To confirm enrichment before amplification, qPCR reactions were performed in triplicate on specific genome test regions by using SYBR Green Super mix (Bio-Rad, Hercules, CA). Positive and negative control regions were the *Rho* promoter and an untranslated region on chromosome 6 (*Untr6*), respectively [7]. The *Untr6* region is 3 Mb downstream of the *Rho* gene.

### Probe preparation and promoter tiling array hybridization

DNAs were amplified using random priming amplification. A fixed sequence of 17 bases containing nine random bases at the 3′ end was used in four linear amplification reactions using Sequenase (USB, Cleveland, OH). Following purification the randomly primed ChIP DNA was amplified for 30 cycles using a fixed primer sequence. The resulting amplified DNA was purified, quantified, and tested by qPCR again to test for conservation of enrichment. Amplified DNA was fragmented and fluorolabeled using the DNA Terminal Labeling Kit (Affymetrix, Santa Clara, CA). Biotinylated probe DNA (1µg) was hybridized to GeneChip Mouse Promoter 1.0R arrays (Affymetrix) overnight at 45 °C. A single array was used for each time point with Pol-II, and a third array was hybridized with non-ChIP total genomic DNA for normalization. A fourth array was used for FIZ1 ChIP DNA at P25. Arrays were hybridized, washed and stained, simultaneously in a GeneChip Fluidics Station (Affymetrix).

### Array scanning and data calculations with Tiling Analysis Software (TAS)

Arrays were scanned with a GeneChip scanner (Affymetrix), and array data files were analyzed using Affymetrix TAS. Array raw input data were normalized using linear scaling to a target intensity (median intensity) of 500, using all perfect matched probes. Two sample analyses were used, with each time point ChIP sample (treatment) compared to the whole genomic chromatin (control). The tail type was one-sided upper with a bandwidth of 400 bp. With the Affymetrix promoter tiling array (probe spacing 35 bp), a suitable bandwidth is 0.5 of the average chromatin fragment size (800 bp) to provide a balance between signal certainty and mapping resolution. (On average this resulted in 22 probes per analysis window for each feature of the array. Higher bandwidths decrease signal p values at the expense of resolution, while lower bandwidths increase resolution at the expense of certainty.) The Wilcoxon signed rank test was applied to log-transformed intensities of the ChIP treatment sample versus the control values for all probes within the analysis window for each feature in the array. Fold enrichment was calculated using the Hodges-Lehmann Estimator (pseudo median) applied to the signal log ratio (log_2_) between the treatment and control signal. Using these ratios, Walsh averages were calculated for each feature in the array, and a final probe signal value was calculated as the median Walsh average. Signal values were exported in bar-file format for interval determination with TAS and visual inspection with Integrated Genome Browser software (IGB, Affymetrix). TAS was also used to determine track intervals. The parameters for track interval determination were: minimum length 300, gap 180, and intermediate thresholds of 2.5 for FIZ1 and 4.0 for Pol-II. TAS and IGB software are available for download from the Affymetrix website. GeneChip array intensity data files (.cel files) and interval track data files (.bed files) were deposited in the Gene Expression Omnibus (GEO) database (GEO accession: GSE19999).

### Determination of gene associations and calculation of prediction metrics

To associate interval regions relative to genes (upstream, in gene, downstream), the track interval results (.bed format) were processed through the TransPath program (Genpathway Inc., San Diego, CA), with upstream and downstream margins from gene ends set to 10 kb.

**Figure 1:**
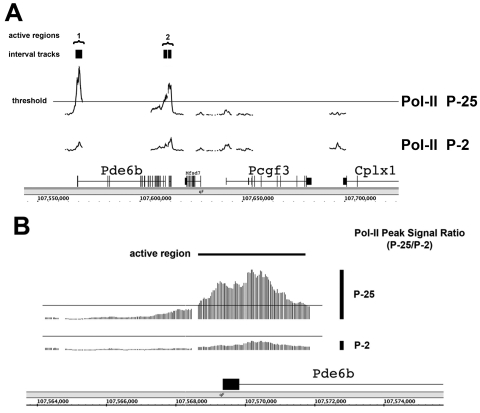
Determination of Pol-II active-region/gene associations and changes to gene activation state for specific genes. RNA-Polymerase-II (Pol-II) ChIP-on-chip data could be examined on any continuous scale from entire chromosomes to single genes. Determination of Pol-II active regions, the association of active regions to genes, and the calculation of specific metrics for each region, required the sequential use of TAS and the TransPath program. **A**: First, TAS generated interval tracks where Pol-II signal levels were above an intermediate threshold. This is illustrated for a region surrounding *Pde6B*, a key marker of photoreceptor terminal maturation. Second, interval track data were processed using TransPath to determine active regions. Active regions were comprised of one or more interval tracks in close proximity. Two active regions are illustrated in the P25 neural retina. **B**: Higher resolution view of the active region surrounding the *Pde6b* TSS. Signals for individual tiling probes (35 bp spacing) are visible. For each active region, TransPath calculated specific metrics, such as the Pol-II peak signal ratio (P25/P2), as illustrated by the vertical black bars indicating signal maxima from the P25 and P2 time point samples. Genes in view: Phosophodiesterase 6b (*Pde6b*), Polycomb group ring-finger 3 (*Pcgf3*), Complexin 1 (*Cplx1*).

Post inspection of the ChIP-on-chip data at internal markers of terminal maturation or any genes of interest was accomplished with the IGB program. *Rho* and *Pde6b* (Phosphodiesterase-6b) provided internal controls as genes previously shown to be inactive at P2 and active at P25 [8,9]. The TransPath program was used to correlate active regions with specific genes and then calculate metrics, such as the Pol-II peak signal ratio (P25/P2). This process is illustrated in Figure 1 for *Pde6b*. *Pde6b* (chromosome 5) encodes phosphodiesterase-6b, a rod-specific subunit of phosphodiesterase that is part of the phototransduction pathway. Mutations of human *PDE6B* cause retinal degeneration through the gradual loss of rod photoreceptors [10].

Interval tracks, determined with TAS, correspond to regions with a probe signal above an intermediate threshold, as described in the previous section. Interval tracks were then processed using the TransPath program to determine Pol-II active regions. Active regions were defined by one or more interval tracks depending on their proximity, as seen in Figure 1A. Tracks less than 5 kb apart were grouped into a single active region. The Pol-II active region spanning the *Pde6b* TSS and exon-1 is also shown at a higher resolution in Figure 1B. Gap regions corresponded to repetitive DNA sequences that were excluded during analysis in TAS, as determined using the Repeat Masker database (which can be viewed in the Genome Browser). Metrics calculated with the TransPath program included the Pol-II peak signal ratio (P25/P2), as illustrated in Figure 1B. The Pol-II peak signal ratio for *Pde6b* was 5.0, predicting an increased association of Pol-II in the mature neural tissue (P25). Coverage on the tiling array extended several thousand bases downstream of the TSS. We have previously demonstrated with ChIP that transcribing Pol-II is detectable deep within the active *Rho* gene [7].

### Quantitative ChIP assays of RNA-Polymerase-II and FLT-3 interacting zinc finger-1 binding

A multitude of photoreceptor genes, known to increase expression from P2 to P25 (i.e., *Rho*, *Pde6b*), provided numerous internal controls to establish confidence in the Pol-II ChIP-on-chip predictions. However, since artifacts may influence the results during ChIP, probe labeling, array hybridization, or washing, we performed quantitative chromatin immunoprecipitation (Q-ChIP) assays for a subset of 36 genes. A selection of genes from the ChIP-on-chip data, representing a full range of Pol-II peak signal ratios (P25/P2), were analyzed by Q-ChIP. Q-ChIP was performed with P2 (immature) and P25 (mature) mouse retina. The retina samples used were different from those used for ChIP-on-chip. Real-time PCR provided a quantitative comparison of the DNA recovered (copy numbers) and thus comparison of the amount of Pol-II or FIZ1 association at target regions [11-13]. Quantitative PCR reactions were performed in triplicate on specific genome test regions, using SYBR Green Super mix (Bio-Rad). The resulting signals were normalized for primer efficiency by carrying out qPCR for each primer pair, using input DNA. Test regions included targets in the *Rho* promoter and an untranslated negative control region on chromosome 6 (Untr6) [7].

Q-ChIP was performed with an antibody to the serine-2-phosphorylated C-terminal repeat domain (CTD) of RNA-Polymerase-II (Pol-II-S2, AbCam Inc., Cambridge, MA). Serine-2 in the Pol-II CTD is phosphorylated in the transcriptionally active form of the enzyme [14,15]. Furthermore, for most genes the target amplicons (about 100 bp) for the Pol-II-S2 Q-ChIP assays were situated downstream of the TSS. FIZ1 Q-ChIP was also performed with anti-bFIZ1 antibody, which is specific for FIZ1 protein in immunoblots of mouse retina and previously qualified for ChIP [7]. Amplicons for FIZ1 Q-ChIP were targeted to regions of peak FIZ1 signal, as predicted from ChIP-on-chip. Amplicons were also placed to avoid repetitive DNA sequences, as determined from mouse genomic data [16], and visualized in the Repeat Masker database with the UCSC Genome Browser.

Threshold cycle number (Ct) values from triplicate real-time PCR assays were transformed using a standard curve of genomic DNA with known copy numbers to obtain precipitated copy numbers for each gene test region. To determine the relative PCR efficiency between different gene regions, triplicate assays were run using nonprecipitated genomic DNA (input). Copy numbers of DNA detected were then normalized for the amount of chromatin input, the proportion of ChIP DNA used for Q-PCR, and then for relative primer efficiency. Final results were scaled as copies of DNA detected per 1,000 genome equivalents of input DNA.

### Gene expression assays—real-time PCR

Specific genes from the ChIP-on-chip data representing a full range of Pol-II peak signal ratios (P25/P2) were analyzed by real-time PCR, using Taqman probe chemistry following the manufacturer’s standard protocol (Applied Biosystems, Foster City, CA). Pre-inventoried assays were selected for most genes with hydrolysis probes (Taqman probes) spanning an exon splice junction. Total RNA was isolated from retinal tissues, using the Absolute RNA Miniprep Kit, and cDNA was synthesized with the AffinityScript qPCR cDNA Synthesis Kit (both from Stratagene-Agilent Technologies, La Jolla, CA)

## Results and Discussion

In this study, mouse neural retina at ages P2 and P25 were analyzed by Pol-II ChIP-on-chip with the Affymetrix Mouse Promoter 1.0R Array. This permitted us to examine the global question: how many genes experience significant activation increases during the terminal maturation of post-mitotic neural progenitors? Most neural progenitor cells in the retina have a fixed cell type by P2. However, this stage is also before the expression of many genes required for actual function of the neural retina. Excellent markers representing this group of genes include phototransduction genes, such as Rhodopsin (*Rho*) and Phosphodiesterase-6b (*Pde6b*). The P2 and P25 ages capture the integration of these neural progenitors into a complex neurosensory tissue. Genes required for photoreceptor and neuron functions are expected to undergo activation during this interval. These should include genes required for synaptogenesis, synaptic function, and ion channels required for neural transmission [17,18].

While the amounts of chromatin required for ChIP-on-chip required large numbers of whole neural retinas, we expected the data set to be mostly sensitive to photoreceptor-derived chromatin as these cells represent about 70% of the mature neural retina. Activation increases in rod-specific genes or synaptic regulatory genes shared with retinal neurons were captured in this analysis. Gene activation increases specific to cones, Müller cells, ganglion cells, and bipolar cells were not captured in our Pol-II ChIP-on-chip analysis based on cell-specific markers for those cells: S-Opsin *(Opn1sw),* M-Opsin *(Opn1mw)* for cones, Glutamine synthetase *(Glu1)* for Müller cells, Glial fibrillary acidic protein *(Gfap)* for ganglion cells, and Protein kinase-C alpha *(Prkca)* for bipolar cells [19,20]. Pol-II signal levels for most of those non-rod genes remained below threshold at both the P2 and P25 analysis points. *Prkca* was just above threshold at P25 but its Pol-II peak signal ratio (P25/P2) was less than 1.8 and was not predicted to experience significant increased activity. Since we are particularly interested in photoreceptors, these properties were useful to predict novel gene activation increases, reducing the resources spent on the pursuit of false positives. Furthermore, we have generated data in a format to share through the genome browsers at UCSC and the National Eye Institute (NEIBank). Instructions for viewing these data are available in the associated supplemental information Appendix 1.

### A substantial percentage of all genes are activated during photoreceptor maturation

After processing with TAS and TransPath, the combined P2 and P25 Pol-II ChIP-on-chip data generated a total of 5,037 Pol-II active regions in the mouse neural retina. Of this total, 2,427 active regions were exclusive to the P25 sample. This result suggested that a substantial number of genes experienced increased activation during terminal differentiation of the neural retina.

Many key genes required for vision, synaptogenesis, and neuron function were captured in this gene set. That fact satisfied the key criterion for successful genome-wide analysis: the enrichment for genes with biologic functions relevant to the model of interest [21-23]. We did not find any of these genes preloaded with Pol-II, as were reported for embryonic stem cells [6], indicating that a major regulatory step for their activation was the recruitment of Pol-II itself. Activated genes displayed Pol-II active regions spanning downstream of their TSS, as illustrated for *Pde6b* (Figure 1B).

### Validation of temporal RNA-Polymerase-II ChIP-on-chip data for predicting increased gene activation state

TAS was used to analyze promoter array data and to calculate Pol-II enrichment signals for all tiling probes, using the linear scaling option. Linear-scaled data were then processed using the TransPath program to calculate additional metrics, including the Pol-II peak signal ratio (P25/P2) for each active Pol-II region. Our aim was to determine a minimal Pol-II peak signal ratio (P25/P2) to accurately predict gene activation increases.

**Figure 2:**
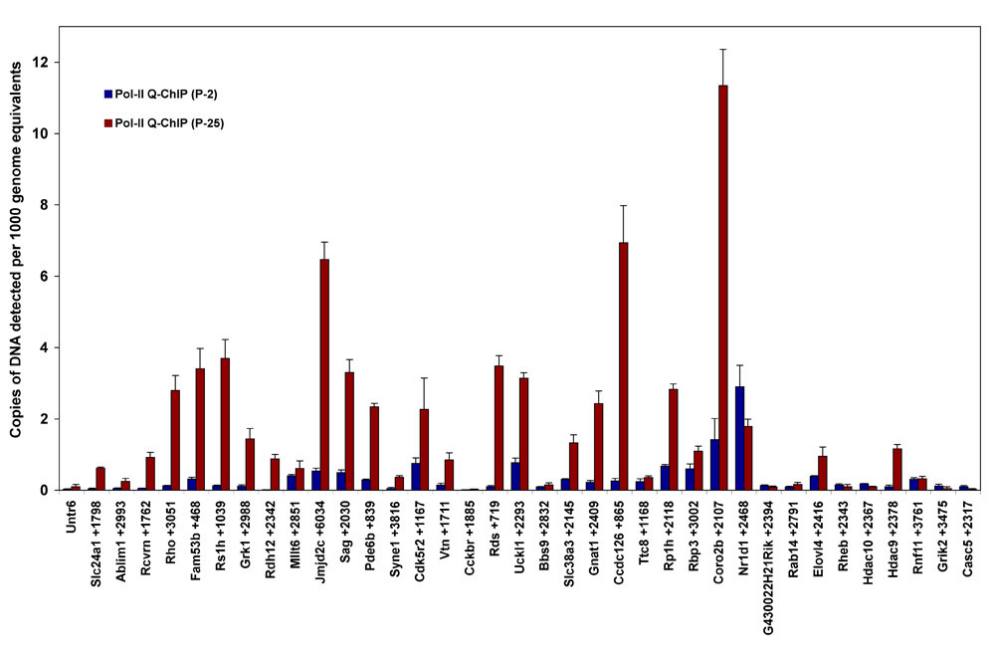
Validation of RNA-Polymerase-II (Pol-II) ChIP-on-chip predictions: changes in activated Pol-II association within genes during terminal maturation of photoreceptors. Q-ChIP assays were performed using an antibody specific for active RNA polymerase-II, which is phosphorylated at serine-2 of the C-terminal repeat domain (Pol-II-S2). Genes assayed represent a full range of ChIP-on-chip Pol-II peak signal ratios (P25/P2) from 14 to 0.5 (Table 1). Pol-II binding is proportional to the amount of target DNA captured by ChIP, graphed as copies of target DNA detected per 1,000 genome equivalents of input genomic DNA. Most assays target in the gene, downstream of the TSS. Results were normalized for chromatin input and PCR efficiency. Error bars indicate standard deviation (n=3). An untranslated region (*Untr6*) on chromosome 6 provided a negative control. The relative position of each PCR target is indicated with the gene symbol. For example, *Slc24a1*+1798 indicates the 5′-end of the PCR target was 1,798 bases downstream of the *Slc24a1* TSS. *Rho*, *Rcvn*, *Pde6b*, and *Sag1* are key markers of photoreceptor-specific gene expression. *Grik2* and *Casc5* were included as genes with ChIP-on-chip Pol-II peak signal ratios <1. Genes: Solute carrier family 24, Na/K/Ca exchanger, member 1 (*Slc24a1*), Actin binding LIM protein1 (*Ablim1*), Recoverin (*Rcvrn*), Rhodopsin (*Rho*), Family with sequence similarity 53, member B (*A930008G19Rik, Fam53b*), Retinoschisis 1 homolog (*Rs1h*), G protein-coupled receptor kinase 1 (*Grk1*), D4, zinc and double PHD fingers, family 3 (*Dpf3*), Retinol dehydrogenase-12 (*Rdh12*), Myeloid/lymphoid or mixed-lineage leukemia 6 (*Mllt6*), Jumonji domain containing 2C (*Jmjd2c*), S-antigen (*Sag*), Phosphodiesterase 6b (*Pde6b*), Spectrin repeat containing, nuclear envelope 1 (*Syne1*), Cyclin-dependent kinase 5, regulatory subunit 2 (*Cdk5r2*), Vitronectin (*Vtn*), Cholecystokinin-2 receptor (*Cckbr*), Peripherin 2 (*Rds*), Uridine-cytidine kinase 1-like 1 (*Uckl1*), Bardet-Biedl syndrome 9 (*E130103I17Rik, Bbs9*), Solute carrier family 38, Na/H -coupled glutamine transporter, member 3 (*Slc38a3*), Transducin alpha (*Gnat1*), Coiled coil domain containing 126 (*6330407D12Rik, Ccdc126*), Tetratricopeptide repeat domain 8 (*Ttc8*), Retinitis pigmentosa 1 (*Rp1h*), Retinol binding protein 3, interstitial (*Rbp3*), Coronin, actin binding protein, 2B (*Coro2b*), Nuclear receptor subfamily 1, group D, member 1 (*Nr1d1*), G430022H21Rik, RAB14, member RAS oncogene family (*Rab14*), Elongation of very long chain fatty acids-like 4 (*Elovl4*), Ras homolog enriched in brain (*Rheb*), Histone deacetylase 10 (*Hdac10*), Histone deacetylase 9 (*Hdac9*), Ring finger protein 11 (*Rnf11*), Glutamate receptor, ionotropic, kainate 2 (beta 2) (*Grik2*), Cancer susceptibility candidate 5 (*Casc5*).

A set of genes with active regions was selected to represent a full range of Pol-II peak signal ratios (P25/P2) derived from the ChIP-on-chip data set (0.5–14). These genes and their Pol-II peak signal ratios are listed in Table 1. Several phototransduction and retinal disease genes were included in this gene set, and additional analysis was performed by Q-ChIP assays, using a second antibody with preference for the actively transcribing form of Pol-II (serine-2 phosphorylated) (see Figure 2). Pol-II Q-ChIP assays also targeted sites deep within the transcribed regions of most of these genes to ensure detection of transcribing Pol-II. Out of 36 genes analyzed by Q-ChIP, most agreed with the predicted changes in Pol-II association, as derived from the ChIP-on-chip analysis; an exception was *G430022H21Rik* and Nuclear receptor subfamily 1 group D member 1 *(Nr1d1).*

**Table 1:** Genes used for validation of ChIP-on-chip predictions.

**Number**	**Gene name**	**Chromosome**	**Pol-II peak signal ratio (P25/P2)**
1	*Slc24a1*	9	14
2	*Ablim1*	19	11.1
3	*Rcvrn*	11	10.9
4	*Rho*	6	10
5	*A930008G19Rik (Fam53b)*	7	8.5
6	*Rs1h*	X	8
7	*Mef2c*	13	6.7
8	*Grk1*	8	6.5
9	*Dpf3*	12	6.5
10	*Rdh12*	12	5.7
11	*Mllt6*	11	5.6
12	*Jmjd2c*	4	5.6
13	*Sag*	1	5.2
14	*Pde6b*	5	5
15	*Syne1*	10	4.9
16	*Cdk5r2*	1	4.9
17	*Vtn*	11	4.4
18	*Cckbr*	7	4.3
19	*Rds*	17	4.1
20	*Uckl1*	2	3.9
21	*E130103I17Rik (Bbs9)*	9	3.8
22	*Slc38a3*	9	3.5
23	*Gnat1*	9	3.5
24	*6330407D12Rik (Ccdc126)*	6	3.2
25	*Ttc8*	12	3
26	*Rp1h*	1	2.9
27	*Rbp3*	14	2.9
28	*Coro2b*	9	2.9
29	*Nr1d1*	11	2.8
30	*G430022H21Rik*	3	2.2
31	*Rab14*	2	2.1
32	*Elovl4*	9	1.8
33	*Rheb*	5	1.8
34	*Hdac10*	15	1.7
35	*Hdac9*	12	1.6
36	*Rnf11*	4	1.3
37	*Grik2*	10	0.9
38	*Casc5*	2	0.5

Genes represent a full range of Pol-II peak signal ratios (P25 / P2). Genes with ratios >1 potentially have increased amounts of Pol-II binding in mature (P25) versus immature (P2) neural retina.

**Figure 3:**
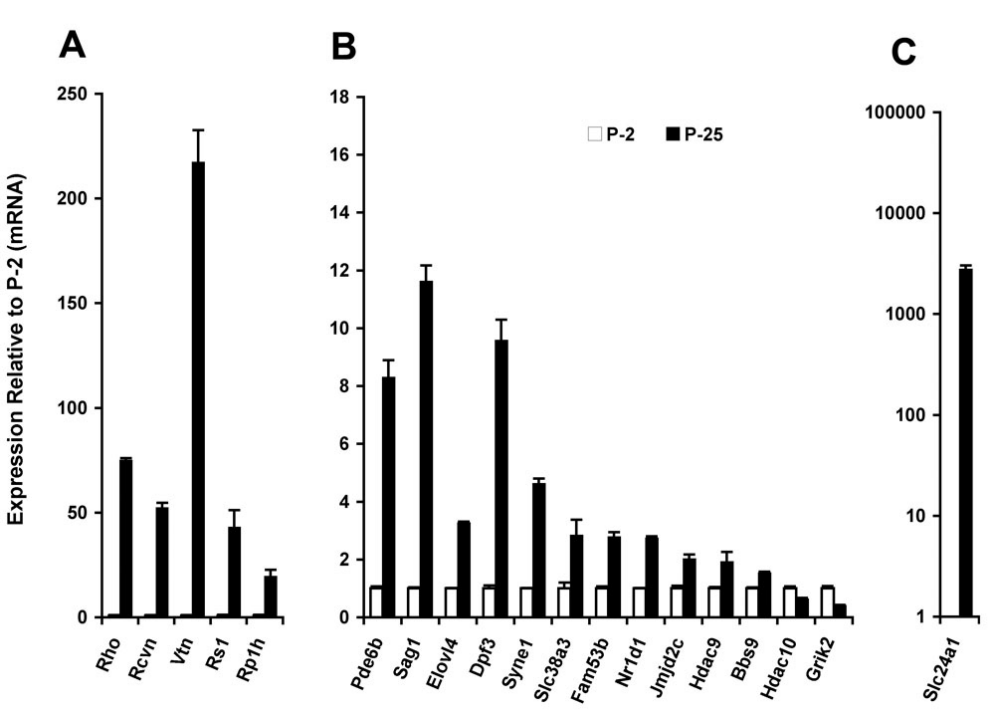
Expression changes (mRNA) of evaluation genes. **A-C**: Relative mRNA concentrations in the mouse neural retina were measured by qPCR (real time) for the developmental ages P2 and P25. Concentrations were normalized to the beta-Actin mRNA concentration. Bars indicate standard deviation for triplicate assays. Taqman chemistry was used for target specificity with hydrolysis probes that span exon junctions. *Rho*, *Rcvn*, *Pde6b,* and *Sag1* are key markers of photoreceptor-specific gene expression. Genes are grouped to account for different scales of relative expression. Most genes had Pol-II peak signal ratios > 1.8, as determined from temporal Pol-II ChIP-on-Chip analysis, except for: *Hdac9* (ratio 1.6), *Hdac10* (ratio 1.7), and *Grik2* (ratio 0.9, Table 1). Genes: Rhodopsin *(Rho),* Recoverin *(Rcvrn),* Retinoschisis 1 *(Rs1),* Phosphodiesterase 6b *(Pde6b),* S-antigen *(Sag),* Elongation of very long chain fatty acids-like 4 *(Elovl4), D4,* zinc and double PHD fingers, family 3 *(Dpf3),* Spectrin repeat containing, nuclear envelope 1 *(Syne1),* Solute carrier family 38, Na/H -coupled glutamine transporter, member 3 *(Slc38a3),* Family with sequence similarity 53, member B *(A930008G19Rik, Fam53b),* Nuclear receptor subfamily 1, group D, member 1 *(Nr1d1),* Jumonji domain containing 2C *(Jmjd2c),* Histone deacetylase 9 *(Hdac9),* Bardet-Biedl syndrome 9 *(E130103I17Rik, Bbs9),* Histone deacetylase 10 *(Hdac10),* Glutamate receptor, ionotropic, kainate 2 *(beta 2) (Grik2),* Solute carrier family 24, Na/K/Ca exchanger, member 1 *(Slc24a1).*

For further evaluation, gene expression assays were completed for 19 genes from the validation gene set, which also represented a full range of Pol-II peak signal ratios. These results are graphed in Figure 3. All of the test genes with Pol-II peak signal ratios >1.8 showed increased expression at P25 based upon neural retina mRNA concentration. Based on the Pol-II Q-ChIP and gene expression results, we concluded that a ChIP-on-chip Pol-II peak signal ratio (P25/P2) ≥1.8 predicts significant increases in the gene activation state with >97% accuracy. This ratio was used for subsequent prediction analysis from the full Pol-II ChIP-on-chip data set.

### Functional analysis of genes that increase activation during photoreceptor maturation

**Figure 4:**
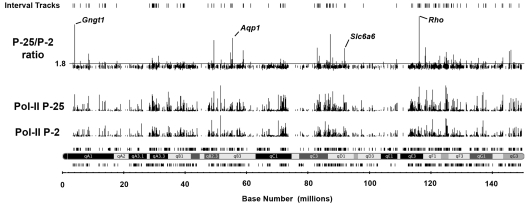
ChIP-on-chip map of changes in RNA-Polymerase-II (Pol-II) association around transcription start sites on chromosome 6 in P2 and P25 mouse neural retina. ChIP was performed using an antibody specific for RNA polymerase-II. ChIP DNA was amplified, labeled, and hybridized onto Affymetrix gene chip mouse promoter tiling arrays (v.1), as detailed in the methods. Probe intensity data were normalized to total genomic DNA (non-ChIP) and the linear scaling format option. Chromosome 6 data are shown here for age P2 (Pol-II P2) and age P25 (Pol-II P25), including a plot of the Pol-II signal ratio (P25/P2 ratio). The horizontal line indicates the Pol-II peak signal ratio (1.8) selected to predict an increased activation state during terminal maturation of photoreceptors. This value was validated through follow-up analysis of the test gene set representing a full range of Pol-II peak signal ratios (Table 1). The locations of many genes displaying activation increases are readily visible. Some examples include Rhodopsin *(Rho),* Transducin-γ subunit *(Gngt1),* Aquaporin *(Aqp1),* and Na: neurotransporter symporter for taurine and β-alanine *(Slc6a6).*

From the Pol-II ChIP-on-chip data, we determined that 1,101 genes (active regions) are predicted to increase activity during terminal maturation of the neural retina, based upon a promoter Pol-II peak signal ratio (P25/P2) ≥1.8. This included genes with or without Pol-II detected above background at the P2 time point. Figure 4 provides a visual illustration of this prediction metric using the ChIP-on-chip Pol-II data for mouse chromosome 6. Several of the genes displaying Pol-II peak signal ratios (>1.8) are indicated, including *Rho*, Transducin gamma chain *(Gngt1)*, aquaporin water channel (*Aqp1*), and Na^+^/Cl^-^-dependent taurine and β-alanine transporter (*Slc6a6*).

Database for Annotation, Visualization and Integrated Discovery (DAVID) annotations were available for 81% of these genes (892), and these genes were analyzed for enrichment according to their gene ontology classifications for biologic processes (GOTERM_BP_ALL database; National Institute of Allergy and Infectious Diseases [NIAID]) [21,24]. There was significant enrichment for biologic processes relevant to this model of neural development, including vision, regulation of transcription, gene expression, neural development/synaptogenesis, neural function, regulation of chromatin, and ion transport. Vision-related genes (55 genes, 6%) included many that encode proteins involved directly in visual transduction and the visual cycle, such as S-antigen/arrestin (*Sag*), retinol-binding protein 3, interstitial (*Rbp3*), phosducin (*Pdc*), ATP-binding cassette A-4 (*Abca4*), recoverin (*Rcvn*), guanine nucleotide-binding protein, α-transducin 1 (*Gnat1*), retinol dehydrogenase 8 (*Rdh8*), retinitis pigmentosa 1 homolog (*Rph1*), *Pde6b*, and *Rho*.

A larger proportion of the genes (157 genes, 18%) have functional annotations involving the regulation of gene expression. These included the photoreceptor-specific transcription factor neural retina leucine zipper (*Nrl*) and other transcription factors, including basic helix loop helix b2 and b3 (*Bhlhb2* and *3*), calmodulin-binding transcription activator 1 (*Camta2*), death effector domain-containing DNA-binding protein 2 (*Dedd2*), D4, zinc and double Phd fingers family 2 and 3 (*Dpf2* and *Dpf3*), eyes absent 3 homolog, *Drosophila* (*Eya3*), Forkhead box O3A (*Foxo3a*), Kruppel-like factor 9 (*Klf9*), myocyte enhancer factor 2d (*Mef2d*), nuclear receptor subfamily 1d1, 1d2, and 4a3 (*Nr1d1*, *Nr1d2*, and *Nr4a3*), and prospero-related homeobox 1 (*Prox1*). The percentage of these genes reported to express in the brain, eye, and retina are 31%, 16%, and 10% respectively.

Functional and tissue enrichment analyses suggested that Pol-II ChIP-on-chip can predict gene activation increases that are functionally relevant to the mature neural retina. This was confirmed by the presence of a substantial number of genes (32) with human homologs that are currently linked to retinal diseases: ATP-binding cassette sub-family A member 4 *(Abca4),* Aryl hydrocarbon receptor interacting protein-like 1 *(Aipl1),* Bardet-Biedl syndrome 4 *(Bbs4),* Bardet-Biedl syndrome 7 *(Bbs7),* Calcium binding protein-4 *(Cabp4),* Calcium channel voltage-dependent alpha1F subunit *(Cacna1f),* Elongation of very long chain fatty acids like 4 *(Elovl4),* Frizzled homolog 4 *(Fzd4),* Guanine nucleotide binding protein, alpha transducing 1 *(Gnat1*), G protein-coupled receptor kinase 1 *(Grk1),* Guanylate cyclase activator 1A and 1B *(Guca1a, Guca1b),* Inosine monophosphate dehydrogenase 1 *(Impdh1),* Jagged 1 *(Jag1),* Potassium channel subfamily V member 2 (Kcnv2), Monogenic audiogenic seizure susceptibility 1 *(Mass1),* Nephronophthisis 4 *(Nphp4), Nrl, Pde6a, Pde6b,* Phosphoglycerate kinase 1 *(Pgk1),* Retinol dehydrogenase 12 *(Rdh12),* Peripherin 2 *(Rds),* Regulation of G-protein signaling 9 (Rgs9), Regulator of G protein signaling 9 binding protein *(Rgs9bp), Rho,* Retinitis pigmentosa GTPase regulator interacting protein 1 *(Rpgrip1),* S-antigen *(Sag),* Tetratricopeptide repeat domain 8 *(Ttc8),* Tubby like protein 1 *(Tulp1),* Unc119 homolog *(Unc119), and* Whirlin *(Whrn;* Retinal Information Network).

### RNA-Polymerase-II ChIP-on-chip prediction of genes mostly activated after age P2

The analysis above included all genes that increase expression from P2 to P25, based upon their associated Pol-II peak signal ratio (P25/P2), regardless of their activation state at the P2 developmental time point. We then focused on genes from the Pol-II ChIP-on-chip data set that were predicted to experience the largest proportion of their activation between P2 and P25. Using the TransPath program we first identified 2,427 genes that were associated with Pol-II active regions above background at P25 but not at P2, as illustrated in Figure 1 for *Pde6b*. These genes were then filtered for those with Pol-II peak signal ratios (P25/P2) ≥1.8, yielding a final list of 863 genes that were predicted to experience the bulk of their activation after age P2. The full list is available in Appendix 2, with each gene’s associated Pol-II peak signal ratio.

Functional annotations were available for 704 of these genes in the DAVID gene ontology database (refer to Table 2). Eighty-nine of the genes (13%) are involved in visual function. Ten of these genes encode proteins of the phototransduction pathway and are known to be mostly activated after P2 in photoreceptors, including *Rho*, Phosducin *(Pdc), Gnat1,* and Recoverin *(Rcvrn)*; 11 genes are involved in synaptic transmission, including Synapse protein 25 *(Snap25),* Acetylcholinesterase *(Ache),* and Solute carrier family 17, sodium-dependent inorganic phosphate cotransporter, member 7 *(Slc17a7)*; 110 genes (16%) have roles in the regulation of gene expression; 106 genes (15%) are involved in signal transduction; and 15 genes (2%) are involved in chromosome organization and biogenesis.

**Table 2:** Pol-II ChIP-on-Chip prediction of genes experiencing most activation after P2.

**Function**	**Count**	**Example genes**
Vision	89	*Sag, Rho, Rpgrip1, Gnat1, Rp1h, Gucy2e*
Phototransduction	10	*Rcvrn, Guca1a, Rho, Gnat1, Pdc, Pdcl3*
Transport	124	*Cacna1f, Kcnj9, Kcnv2, Slc25a35, Slc23a3*
Regulation of transcription	92	*Ahr, Ankrd33, Cyr2, Nr4a3, Pbx3, Eya3*
Gene expression	110	*Smad4, Dpf3, Jmjd2c, Nr1d2, Rxra*
Neuron development	11	*Nfasc, Ntng2, Syngap1, Cacna1f*
Nervous system development	21	*Map2k1, Cln8, Ablim1, Rora, Lrp2*
Ion transport	33	*Slc38a3, Slc24a1, Clcn3, Kcnj9*
Signal transduction	106	*Gngt1, Gnb1, Gucy1a3, Rp1h, Grk1*
Synaptic transmission	11	*Ache, Cln8, Snap25, Gabra1, Epas1*
Chromatin modification	9	*Jmjd2c, Sap18, Cbx8, Sirt2*
Chromatin organization	15	*Smarca2, Jmjd2b, Jmjd2c, Jarid1b*
CNS development	6	*Cln8, Rora, Sept4, Pex5, Top2b, Lrp2*

To select for genes predicted to experience most of their activation increase after age P2, the criteria were: Pol-II signal levels below background threshold at P2, and Pol-II peak signal ratios (P25/P2) ≥1.8. Functional clustering analysis was performed using the DAVID gene ontology database. Complete lists of all genes, with their associated Pol-II peak signal ratios (P25/P2), may be found in Appendix 2.

### FIZ1 recruitment to genes activated during photoreceptor maturation

FIZ1 is a zinc-finger protein that is recruited to the promoter complex of several photoreceptor genes that become active during terminal maturation of the neural retina. FIZ1 can interact directly with two transcription factors, cone-rod homeobox (CRX) and NRL, that are required for activating photoreceptor genes [7,8]. While FIZ1 expression is ubiquitous in adult mammals, FIZ1 protein content increases tenfold during postnatal maturation of the mouse neural retina [8]. While all of FIZ1’s functions and interactions remain to be elucidated, the availability of a ChIP-qualified antibody made it possible to use FIZ1 as an additional biomarker for genes becoming activated in the adult retina.

**Figure 5:**
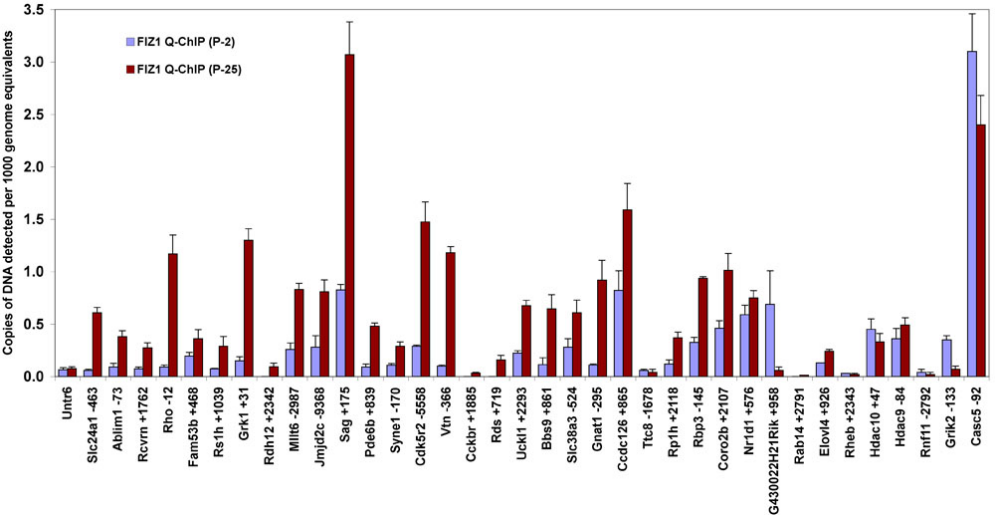
Comparison of FLT-3 interacting zinc finger 1 (FIZ1) recruitment to gene promoter complexes during terminal maturation of photoreceptors. ChIP was performed using an antibody specific for the FIZ1 protein. Protein binding is proportional to the amount of target DNA captured by ChIP and is shown as copies of target DNA detected per 1,000 genome equivalents of input DNA. Results were normalized for chromatin input and PCR efficiency. Error bars indicate standard deviation (n=3). An untranslated region (*Untr6*) on chromosome 6 provided a negative-binding control. The 5′ end location of each PCR target is indicated with each gene name. For Example, *Slc24a1*-463 indicates that the qPCR target was 463 bases upstream of the *Slc24a1* TSS. *Rho*, *Rcvn*, *Pde6b*, and *Sag1* are key markers of photoreceptor-specific gene expression. *Grik2* and *Casc5* were included as genes with ChIP-on-chip Pol-II peak signal ratios <1.0. Genes: Solute carrier family 24, Na/K/Ca exchanger, member 1 (*Slc24a1*), Actin binding LIM protein1 (*Ablim1*), Recoverin (*Rcvrn*), Rhodopsin (*Rho*), Family with sequence similarity 53, member B (*A930008G19Rik, Fam53b*), Retinoschisis 1 homolog (*Rs1h*), G protein-coupled receptor kinase 1 (*Grk1*), D4, zinc and double PHD fingers, family 3 (*Dpf3*), Retinol dehydrogenase-12 (*Rdh12*), Myeloid/lymphoid or mixed-lineage leukemia 6 (*Mllt6*), jumonji domain containing 2C (*Jmjd2c*), S-antigen (*Sag*), Phosphodiesterase 6b (*Pde6b*), Spectrin repeat containing, nuclear envelope 1 (*Syne1*), Cyclin-dependent kinase 5, regulatory subunit 2 (*Cdk5r2*), Vitronectin (*Vtn*), Cholecystokinin-2 receptor (*Cckbr*), Peripherin 2 (*Rds*), Uridine-cytidine kinase 1-like 1 (*Uckl1*), Bardet-Biedl syndrome 9 (*E130103I17Rik, Bbs9*), Solute carrier family 38, Na/H -coupled glutamine transporter, member 3 (*Slc38a3*), Transducin alpha (*Gnat1*), Coiled coil domain containing 126 (*6330407D12Rik, Ccdc126*), Tetratricopeptide repeat domain 8 (*Ttc8*), Retinitis pigmentosa 1 (*Rp1h*), Retinol binding protein 3, interstitial (*Rbp3*), Coronin, actin binding protein, 2B (*Coro2b*), Nuclear receptor subfamily 1, group D, member 1 (*Nr1d1*), G430022H21Rik, RAB14, member RAS oncogene family (*Rab14*), Elongation of very long chain fatty acids-like 4 (*Elovl4*), Ras homolog enriched in brain (*Rheb*), Histone deacetylase 10 (*Hdac10*), Histone deacetylase 9 (*Hdac9*), Ring finger protein 11 (*Rnf11*), Glutamate receptor, ionotropic, kainate 2 (beta 2) (*Grik2*), Cancer susceptibility candidate 5 (*Casc5*).

Out of 863 genes predicted to experience most of their activation after P2, based on Pol-II binding (previous section), 243 genes (28%) scored positive for FIZ1 association at their promoter regions in adult (P25) neural retina. A full list of these genes and their locations may be found in Appendix 3. FIZ1 ChIP-on-chip predictions were confirmed by additional FIZ1 Q-ChIP assays, using a second antibody for FIZ1 and the same set of genes analyzed by Pol-II Q-ChIP. FIZ1 Q-ChIP results for 36 genes at the P2 and P25 developmental stages confirmed the recruitment of FIZ1 to most of the genes experiencing increases in Pol-II-Ser2 association during terminal maturation (see Figure 5).

Functional clustering analysis was possible for 162 of the genes that had sufficient annotations in the DAVID database. These are summarized in Table 3: 20% have roles in vision, 22% in signal transduction, 22% in gene expression, and 26% in transport processes. FIZ1 active areas were associated with 15 currently known retinal disease genes: *Bbs7*, *Bbs9*, *Elovl4*, *Gnb1*, *Gnat1*, *Gngt1*, *Grk1*, *Jag1*, *Sag*, *Pde6a*, *Pde6b*, *Rho*, *Rdh12*, *Rp1h*, and *Rpgrip1*. FIZ1 Q-ChIP assays for eight of those genes (*Bbs9*, *Elovl4*, *Gnat1*, *Grk1*, *Sag*, *Pde6b*, *Rho*, and *Rp1h*) confirmed an increased association of FIZ1 at P25 versus P2 (Figure 5). While the precise role of FIZ1 in the formation of these activation complexes is not known at this time, evidence to date suggests that FIZ1 recruitment to photoreceptor gene promoters is dependent on DNA-binding pioneer transcription factors, such as CRX and NRL [7,8]. Indeed, expression for many of the genes were reported in green fluorescent protein (GFP)-sorted rod photoreceptors, and their expression is diminished in NRL^(−/−)^ mice [25], including *Gnat1, Rho, Grk1, Jag1, Pde6b, Gnb1, Rdh12, Rd1h, Sag, Rcvrn,* Interphotoreceptor matrix proteoglycan 1 *(Impg1),* WD repeat domain 17 *(Wdr17),* Nucleoredoxin-like 1 *(Nxnl1),* Stomatin *(Stom),* Membrane protein palmitoylated 4 *(Mpp4),* and Rho GTPase activating protein 24 *(Arhgap24)*.

**Table 3:** Genes activated in maturing neural retina that recruit FIZ1 to promoter complexes.

**Function**	**Count**	**Example genes**
Vision	32	*Rho, Rcvrn, Sag, Pde6b, Rp1h*
Phototransduction	5	*Rcvrn, Rho, Gnb1, Gnat1, Pdc*
Transport	42	*Rcvrn, Clcn3, Slc38a3, Kctd7*
Regulation of transcription	27	*Ahr, Mier1, Pbx3, Rora, Ablim1*
Gene expression	36	*Jmjd2c, Ahr, Pprc1, Srct1, Klf9*
Ion transport	12	*Clcn3, Slc38a3, Slc24a1, Kctd7*
Neuron development	4	*Nfasc, Mtap2, Ablim1, Rtn4*
Nervous system development	9	*Ipmk, Map2k1, Nfasc, Sep4*
Signal transduction	36	*Gngt1, Gnb1, Rgs9bp, Pde6b*
Synaptic transmission	3	*Snap25, Park2, Slc17a7*
Chromatin organization	4	*Jmjd2c, Ercc1, Nap1l2, Bnip3*

Criteria were: Pol-II peak signal ratio (P25/P2) ≥1.8, and FIZ1 peak signal above background threshold. Functional clustering was performed using the DAVID gene ontology database. A complete list of all genes may be found in Appendix 3.

### Novel gene activations predicted by temporal RNA-Polymerase-II ChIP-on-Chip analysis

Expression microarray studies of mouse retinal development have revealed hundreds of genes that increase expression during terminal maturation [26,27]. Those studies revealed important information regarding gene regulation during retinal development and expanded the list of candidate genes for numerous retinal diseases. Even so, many gene expression changes await discovery for two reasons. First, different array platforms detect many gene expression changes not captured by the other platform [26]. Second, hybridization of a complex probe population limits the ability to see changes in the lower abundance transcripts.

Pol-II ChIP-on-chip examines relative changes in Pol-II association; therefore, it had the potential to detect the activation of additional genes. Even a few hundred additional gene activation events would be valuable data to supplement those already revealed by expression microarrays. To confirm the potential of Pol-II ChIP-on-chip to find additional genes activated during terminal maturation of the retina, we compared our Pol-II ChIP-on-chip gene activation predictions (Pol-II peak signal ratio P25/P2≥1.8) with two excellent sets of published expression microarray data [26,27]. Because of different array platforms and differences in the principal detection method (mRNA versus Pol-II), a substantial number of gene activation predictions were unique to all three data sets. From a three-way comparison, 820 activation events were unique to the Pol-II ChIP-on-chip-predicted data set. The three gene lists that were compared and the ChIP-on-chip unique list are available in Appendix 4.

Functions of the ChIP-on-chip unique genes are summarized in Table 4. A substantial number were associated with vision, including *Guca1b,* Retinol dehydrogenase-8 *(Rdh8),* Guanylate cyclase membrane type-e *(Gucy2e),* Retinal degeneration-3 *(Rd3, 3322402L07Rik),* Metabotropic glutamate receptor-6 (*Grm6*), *Rdh12, Bbs4, Aipl1*, and *Cacna1f. Aipl1* and *Rdh12* activation increases were previously detected by Akimoto et al. [25] with expression microarrays, but only after GFP sorting to isolate rod photoreceptors.

**Table 4:** Functional annotation analysis (DAVID) of novel gene activations, predicted by Pol-II ChIP-on-Chip.

**Function**	**Count**	**(%)**
Regulation of transcription	90	14
Gene expression	118	18
Visual perception	13	2
System process (vision)	26	4
Transport	92	14
Phototransduction	3	1
Signal transduction	80	12
Nervous system development	18	3
Neuron development	9	1
Synaptic transmission	5	1
Chromosome organization and biogenesis	12	2
Chromatin modification	5	1
Ion transport	17	3
Central nervous system development	5	1

Gene activations predicted by Pol-II ChIP-on-Chip, which are additional to those reported by two previous microarray studies. The full list of genes (820) and list comparisons are provided in Appendix 4. Of the 820 genes, 463 had sufficient annotations for functional clustering, and the results are summarized in this table.

Pol-II ChIP-on-chip analysis was particularly sensitive to detecting increased activation of genes involved in transport processes and ion transport. These included many solute carrier family proteins, such as *Slc26a6*, *Slc41a1*, *Slc4a10*, and *Slc4a7*. Also enriched were cellular genes encoding subunits of mitochondrial ATPase: *Atp1a3*, *Atp8a1*, *Atp5d*, *Atp5b*, and *Atp5k*. *Atp1a3* mutations are associated with rapid onset dystonia parkinsonism [28]. Disruption of *Slc4a10* (sodium bicarbonate transporter) was recently associated with complex partial epilepsy, and mice lacking Slc4a7 develop degeneration of sensory receptors of the eye and inner ear, as in Usher syndrome [29]. These neurosensory associations emphasize that these transport families are important for functionality and represent a large and additional pool of genes to explore during maturation of the neural retina.

### Novel candidates for genes involved in retinal maturation and function

From the ChIP-on-chip unique predictions of genes showing increased activation after P2, ten have human homologs that are linked to human retinal diseases [30-40]: *AIPL1, BBS4, CACNA1F, RDH12*, Retinitis Pigmentosa 1 (*RP1*), Glutamate Receptor, Metabatropic 6 (*GRM6*), *GUCA1B*, Retinal Degeneration 3 (*RD3*), and Ceroid-Lipofuscinosis, Neuronal-5 and −8 (*Cln5, Cln8*). This demonstrates that the ChIP-on-chip predictions should contain additional genes necessary for normal retinal function. Some are related to other known visual function genes, and we suggest they are good candidates for further exploration. *Rdh8* is a member of the retinol dehydrogenase gene family, and another family member, *RDH12,* is linked to retinal degenerations. Phosducin-like 3 protein (*Pdcl3*) and other members of this gene family (*Pdcl1* and *2*) appear to be co-chaperones with the cytoplasmic chaperonin complex. Phosducin itself has a visual role as a chaperone for G protein β-γ subunits as they are translocated between photoreceptor outer and inner segments during light adaptation [41].

Approximately 250 of the genes do not have well defined functions and also represent a potential source of novel genes required for maturation and function of the neural retina. We examined the chromosome locations of their human homologs and found that several are located within the mapped regions of unidentified retinal diseases (Table 5). *SLC25A33* is located in the region for LCA9 [42] and encodes for a mitochondrial carrier family member with relatively high expression in the eye (qPCR) compared to other members of this large gene family. It is also expressed at relatively higher levels in neural tissues, including the spinal cord and the cerebellum, while its expression in peripheral tissues is quite low [43]. Lysophosphatidylcholine acyltransferase 1 (*LPCAT1*) is one of three transmembrane LPCAT family members that transfer fatty acids from acyl-CoA to lysophosphatidylcholine [44]. Human *LPCAT1* is in the mapped region for dominant macular dystrophy-3 [45]. Several other genes involved in lipid homeostasis and transport have been linked to retinal diseases, including dominant cone-rod dystrophy [46] and recessive abetalipoproteinemia [47].

**Table 5:** Some examples of gene activation increases predicted by Pol-II ChIP-on-Chip, which have human homologs located in mapped intervals of unidentified retinal diseases.

**Human disease (location)**	**Human gene name**	**Pol-II peak signal ratio mouse homolog (P25/P2)**	**mRNA expression P2 versus P25 mouse neural retina (±SD, n=3)**		**mRNA expression normal versus Rd1 mouse neural retina (±SD, n=3)**	
			**P2**	**P25**	**Normal (P33)**	**Rd1 (P33)**
LCA9(1p36)	*SLC25A33*	2	1.00±0.07	7.1±0.1†	1.00±0.06	0.49±0.01†
MCDR3(5p15.33-p13.1)	*LPCAT1*	2.2	1.00±0.03	3.5±0.4†	1.00±0.01	0.55±0.05†
MDDC, CYMD (7p21-p15)	*CCDC126*	3.2	1.0±0.1	26.1±0.2†	1.00±0.04	0.09±0.03†
CORD4(17q) ‡	*ARL4D*	3.6	1.0±0.2	127±11†	1.00±0.04	0.12±0.01†

Pol-II peak signal ratios (P25/P2) are derived from ChIP-on-Chip data and predicted gene activation (≥1.8). Relative gene expression was then examined by real time PCR (Taqman) comparing P25 and P2 normal retina. Gene expression was also compared between normal retinas (P33) and *Rd1* retinas (P33) that had lost >99.7% of their rod-photoreceptors. † p<0.001, *t*-test. LCA9 (recessive Leber congenital amaurosis, Type 9); MCDR3 (dominant macular dystrophy-3); MDDC, CYMD (dominant macular dystrophy cystoid); CORD4 (cone rod dystrophy) ‡ location by estimation, not yet mapped.

The *CCDC126* gene encodes for a protein of unknown function: coiled-coil domain-containing 126 . The human gene falls in the mapped interval reported for autosomal dominant cystoid macular dystrophy [48]. *ARL4D* (*Arf4l*) encodes for ADP-ribosylation factor-like 4D [49]. ARL family members are related to the ADP-ribosylation factor (ARF) family of proteins. ARF proteins have important roles in intracellular traffic, and the ARL proteins are a guanosine triphosphates (GTP)-binding protein family. Another member from this family, *ARL6*, was identified as the gene for Bardet-Biedl syndrome-3 (BBS-3), a disorder that includes retinopathy, learning disabilities, obesity, and renal and cardiac malformations [50].

Follow-up gene expression analysis confirmed that the expression of all four genes is increased during maturation of the neural retina (Table 5). *Arl4d* and *Ccdc126* demonstrated the largest maturation-related expression increases of 26 fold and 127 fold, respectively. *Slc25a33* and *Lpcat1* expression increased 3.5 fold and sevenfold, respectively. The expression of all four genes was diminished in mature *Rd1* mouse neural retina compared to normal retina. *Rd1* neural retinas were compared at age P33, and loss of over 99.7% of rod photoreceptors and 50% of cone photoreceptors was confirmed by gene expression analysis of *NRL and M-opsin* (data not shown). Relative expression of *Slc25a33* and *Lpcat1* was reduced by half in *Rd1* retinas, indicating relatively high expression in photoreceptor neurons. Relative expression in photoreceptors was much higher for *Arl4d* and *Ccdc126*; their overall retinal expression was diminished by 90% in *Rd1* retinas compared to normal retinas.

### RNA-Polymerase-II ChIP-on-chip analysis of the Rhodopsin promoter

An additional benefit of high-resolution ChIP-on-chip data are the ability to visualize differences in Pol-II association along adjacent portions of the DNA sequence. This geographic interpretation can reveal mechanistic clues pertaining to specific gene promoters or alternate promoters. Regions of maximal Pol-II signal can reveal promoter sequences that are brought into close proximity within the DNA/protein complex of the enhanceosome. Upstream enhancers and the TSS can come into close proximity through interactions of proteins in the activating promoter complex, resulting in the formation of chromatin loops [51]. In cell culture estrogen receptor activates the *CTSD* gene with the concomitant formation of a chromatin loop, which brings a distal upstream enhancer region into close proximity to the TSS. As a result, Pol-II maps by ChIP at both the distal enhancer and the TSS of the activated Cathepsin D *(CTSD)* gene [52]. A detailed examination of our *Rho* Pol-II ChIP-on-chip data suggested that similar loop mechanisms can also exist in native tissues.

**Figure 6:**
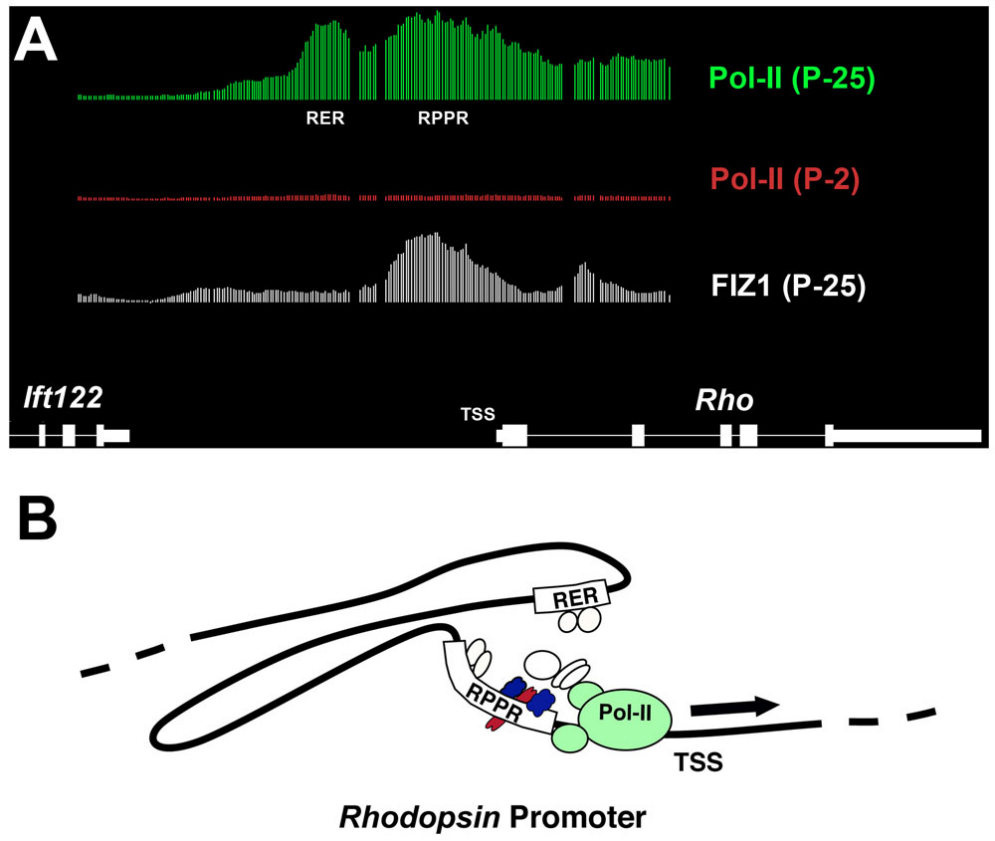
RNA-polymerase-II (Poll-II) associations at the activated rhodopsin gene (*Rho*). **A**: Pol-II ChIP-on-chip data viewed with the IGB program around the *Rho* transcription start site (TSS), including the rhodopsin proximal promoter region (RPPR) and the upstream rhodopsin enhancer region (RER). The *Rho* gene was not preloaded with inactive Pol-II in P2 neural retina before expression in maturing photoreceptors. Two regions of maximum Pol-II signal corresponded to the RPPR and RER regions of the promoter. The FIZ1 ChIP signal maximum also corresponded to the RPPR. **B**: While the RPPR is sufficient for gene activation, the RER is required for maximum activity. Transcription factors shown to enhance *Rho* expression can bind to sequences in the RER, about 2,500 bp upstream of the RPPR. The Pol-II ChIP-on-chip pattern is consistent with a DNA loop model where distal enhancer regions (the RER) become integrated with the gene’s RPPR in the full enhanceosome complex.

High-resolution plots of Pol-II association for *Rho* are presented in Figure 6A. Two Pol-II signal maxima were noted to correspond to the Rhodopsin proximal promoter region (RPPR) and the upstream Rhodopsin enhancer region (RER) [53-57]. Both regions bind nuclear proteins from neural retina and are involved in the activation and enhancement of *Rho* promoter activity. The RPPR is essential for activation by the transcription factors NRL and CRX [58-60]. Several DNA sequence elements in this 500-bp region bind transcriptional proteins in vitro. These include the NRL response element that is present in several rod-specific genes: *RHO*, *PDE6B*, *a-PDE*, and *GNAT1* [59-70]. NRL synergistically co-activates the *Rho* promoter with CRX, and these two proteins directly interact [58,66,71]. Several homeobox transcription factors, Retina and anterior neural fold homeobox−2 (RAX2), Retina and anterior neural fold homeobox (RAX), and Cone-rod homeobox (CRX), can bind to the Ret-1/photoreceptor conserved element-1 (PCE-1) DNA-sequence element of the RPPR and also enhance activity of the promoter [67,72,73]. NRL and CRX can directly bind FIZ1. FIZ1 ChIP-on-chip resulted in a maximum FIZ1 signal corresponding to this NRL- and CRX-binding region (Figure 6A).

While the RPPR is sufficient for activation, maximal expression of the rhodopsin gene requires the RER, which is a conserved region (about 100 bp) found 1.5–2 kb upstream of the TSS, depending on the species [63]. The bimodal distribution of Pol-II association at the *Rho* promoter suggests the formation of an activating chromatin loop between the RER and RPPR regions in vivo. (Figure 6B)

### Conclusions

We have analyzed the activation of thousands of genes during terminal maturation of a neural tissue using ChIP-on-chip to map Pol-II association around TSSs. Relative increases in Pol-II binding could accurately predict genes activated during maturation of photoreceptors in the retina. Pol-II ChIP-on-chip detected the activation of several hundred genes in addition to those known from previous expression microarrays analysis. These represent a substantial and novel source of candidate genes that support retinal function. Many of the genes also have human homologs located within the mapped intervals of currently unidentified human retinal diseases, which we continue to explore. Unlike many genes in embryonic stem cells, genes activated during terminal maturation of photoreceptors did not appear to be preloaded with inactive Pol-II. High-resolution mapping of Pol-II association can also reveal gene-specific promoter activation mechanisms. One example is the involvement of the upstream enhancer region in the activated *Rho* promoter complex. Genome-wide maps of Pol-II association around TSSs in a mammalian retina (mouse) provide a new resource for correlation to expression data. These maps are also viewable in the UCSC Genome Browser and EyeBrowse as a shared resource.

## Appendix 1. Supplemental Genome Browser Guide.

To access the data, click or select the words “Appendix 1.” This will initiate the download of a (pdf) archive that contains the file.

## Appendix 2. Genes predicted to experience most of their activation after P-2.

To access the data, click or select the words “Appendix 2.” This will initiate the download of a (pdf) archive that contains the file.

## Appendix 3. Genes activated after P2, with Pol-II peak signal ratios ≥ 1.8, that recruit FIZ1.

To access the data, click or select the words “Appendix 3.” This will initiate the download of a (pdf) archive that contains the file.

## Appendix 4. Novel gene activation increases predicted by Pol-II ChIP-on-Chip

Genes. To access the data, click or select the words “Appendix 4.” This will initiate the download of a (pdf) archive that contains the file.
